# Evaluation of Artificial Intelligence-Assisted Video Monitoring for Inpatient Fall Prevention: A Retrospective Matched Cohort Study

**DOI:** 10.3390/healthcare14182999

**Published:** 2026-09-14

**Authors:** Dong-suk Lee, Young-Ju Kim, Hee-won Park, Seung-ok Choi, Gyeong-Nam Lee, JiHoon Park, MoonKi Choi

**Affiliations:** 1 College of Nursing, Kangwon National University, Chuncheon 24341, Republic of Korea; ds1119@kangwon.ac.kr; 2 Department of Statistics, Kangwon National University, Chuncheon 24341, Republic of Korea; ykim7stat@kangwon.ac.kr; 3 Department of Rehabilitation Medicine, Kangwon National University, Chuncheon 24341, Republic of Korea; stefen@hanmail.net; 4 Department of Rehabilitation Medicine, Kangwon National University Hospital, Chuncheon 24289, Republic of Korea; 5 Department of Medical Innovation, Kangwon National University Hospital, Chuncheon 24289, Republic of Korea; muse104@naver.com; 6 Department of Nursing Science, Hallym Polytechnic University, Chuncheon 24210, Republic of Korea; leekn@hanmail.net; 7 Accredited Corporate R&D Center, Geomexsoft, Chuncheon 24461, Republic of Korea; if@geomex.co.kr

**Keywords:** falls, inpatients, intelligent systems, safety management, video-assisted techniques and procedures

## Abstract

**Highlights:**

**What are the main findings?**

- Artificial intelligence-assisted video monitoring was clinically evaluated using a 1:1 matched cohort of hospitalized patients.
- Fall incidence was lower after implementation, but no statistically significant reduction was observed.

**What are the implications of the main findings?**

- The findings highlight both the potential and limitations of artificial intelligence-assisted video monitoring for inpatient fall prevention.
- Monitoring systems should evolve beyond fall detection to identify fall-risk behaviors and environmental cues for proactive fall prevention.

**Abstract:**

**Background/Objectives**: Effective strategies to prevent inpatient falls are essential for reducing fall-related injuries, mortality, length of hospital stay, and healthcare costs. Although advanced technologies have increasingly been adopted for fall prevention, evidence regarding effectiveness in real-world clinical settings remains limited. This study evaluated the effect of implementing an artificial intelligence-assisted video monitoring system on the incidence of inpatient falls and fall-related injuries. **Methods**: This retrospective matched cohort study used electronic medical record data from a tertiary hospital in C city, South Korea. The system was implemented in January 2022. Patients admitted between 2020 and 2021 comprised the non-exposed group, whereas those admitted between 2023 and 2024 comprised the exposed group. Nearest neighbor propensity score matching based on age, sex, the number of diagnoses, and the number of ward days was performed to create comparable groups. Fall incidence rates per 1000 patient-days were calculated, and Firth’s penalized likelihood logistic regression and Cox proportional hazards regression with robust errors were conducted. **Results**: Propensity score matching yielded a 1:1 matched sample of 3002 cases per group. The fall incidence rate was 1.017 per 1000 patient-days in the exposed group, lower than 1.286 in the non-exposed group. However, penalized likelihood logistic regression and Cox proportional hazards regression revealed no statistically significant effect of artificial intelligence-assisted video monitoring on fall reduction. **Conclusions**: Artificial intelligence-assisted video monitoring was associated with a lower fall incidence, but no statistically significant effect was identified. These findings highlight the potential and limitations of artificial intelligence-assisted video monitoring for inpatient fall prevention and underscore the need for further research to enhance its clinical utility.

## 1. Introduction

Patient falls are the most common safety-related adverse event in hospitals, thereby posing a significant and persistent problem. According to a Korean report, falls account for 47.2% of all patient safety incidents [1]. In the United States, an estimated one million falls occur annually in hospitals [2]. Inpatient falls can result in injuries such as contusions, lacerations, fractures, and bleeding, which subsequently lead to the need for additional treatment, prolonged lengths of hospital stay, increased mortality, higher healthcare costs, increased risk of litigation, and compromised quality of care [3]. Research has reported direct medical costs associated with falls of up to $50 billion [2]. Despite considerable heterogeneity among the guidelines, hospitals employ various strategies to mitigate falls, including intentional rounding to meet patient needs, sitters providing one-to-one surveillance, patient education, and environmental modifications, and physical restraints as a “last resort” [4].

In recent years, significant advancements have been made in technologies designed to detect and recognize falls [5]. In addition, remote data collection techniques and wireless communication technologies like Bluetooth have facilitated this progress [5]. These technologies typically collect data via wearable or non-wearable (environmental) sensors, such as cameras, accelerometers, and gyroscopes, and then detect and recognize falls by estimating pose and position. Commonly referred to as video monitoring or video surveillance, patient safety surveillance through vision-based fall detection systems using cameras is being increasingly utilized in clinical settings because of its numerous advantages, including the ability to visually verify events and capture detailed contextual information surrounding falls [6]. Although wearable or portable cameras can also collect visual data, fixed-camera video monitoring is considered particularly advantageous in hospital settings because of its ease of use and management, despite ongoing privacy concerns [7]. Fixed-camera video monitoring reduces the burden of individual device management for patients, such as charging, compliance with wearing, and network setup, and enables the simultaneous monitoring of patient conditions and hospital room environments. This approach is particularly effective in multibed hospital rooms [7,8,9].

Previous studies have detailed attempts to prevent falls through video monitoring, primarily utilizing a remote video-monitoring approach [7,10,11]. The key components of such monitoring include video surveillance and the use of a “virtual sitter” (or telesitter), who remotely monitors patients to ensure their safety. In this approach, virtual sitters are stationed at a centralized location, where they observe 12 to 15 patients in real time. When a fall risk is identified, virtual sitters intervene either by communicating directly with the patient via a speaker or by notifying the unit nurse [7,10,11]. Hogan Quigley et al. (2022) reported a 14% reduction in fall rates and a 6% decrease in fall-related injuries following the implementation of remote video monitoring, along with cost-saving benefits [10]. Ergai et al. (2024) documented a reduction in injurious falls, and noted that nurses, who are the primary users of the system, positively evaluated its acceptability and usefulness [11]. However, communication challenges between virtual sitters and unit nurses have been identified as a major barrier [7,11], thus underscoring the fundamental limitation of virtual sitters, which rely on remote interaction.

In some cases, technology-based systems have been developed to automate functions traditionally performed by virtual sitters, including the detection of patient falls [9,12]. Artificial intelligence (AI)-assisted video monitoring can identify patients’ pose and position, thereby detecting falls and notifying nurses. Recent advances in computer vision and deep learning have enabled automated fall detection from video data. For example, Wu et al. (2023) developed a weakly supervised dual-modal network that detected falls from video using Red-green-blue (RGB) and optical-flow information without requiring fine-grained temporal annotations [13]. This strategy appears promising, as it enables continuous monitoring and facilitates rapid intervention by nursing staff while reducing reliance on dedicated human monitoring personnel. However, human virtual sitters may recognize contextual cues and early warning signs that current AI systems may not reliably detect. Thus, whether AI-assisted video monitoring can achieve clinically meaningful fall prevention effects comparable to those of human-assisted monitoring requires evaluation in real-world clinical settings.

More broadly, AI technologies are increasingly being integrated into healthcare, with recent advances in multimodal foundation models expanding their potential applications across a wide range of clinical tasks, including early diagnosis, disease prediction, customized treatment, and clinical decision support [14]. Nevertheless, the increasing technological capability of AI does not necessarily translate into effective clinical implementation. Challenges related to data quality and standardization, model generalizability, and interpretability remain important considerations, while the clinical effectiveness of AI technologies requires evaluation in real-world healthcare settings. Recent research has emphasized that effective translation of AI-enabled technologies from technological innovation into clinical and public health practice is essential for realizing their potential benefits [15]. Hospitals also play an important role in connecting technological development with clinical application and evaluation of AI-enabled medical devices [16]. Accordingly, evaluating AI-assisted monitoring in clinical environments is essential to determine whether its technological capabilities translate into improved patient safety outcomes.

This study evaluated the effect of AI-assisted video monitoring on inpatient falls in a tertiary hospital in the Republic of Korea. The system provides continuous video monitoring through cameras installed in hospital rooms. When AI detects a fall, an alert is sent to the nurses’ station, enabling nurses to monitor patients and room environments in real time and respond without communication delays, thereby facilitating more efficient fall management. Despite the growing adoption of such technologies, empirical evidence on their effectiveness in real-world hospital settings remains limited. Through a comparison of fall outcomes before and after the implementation of AI-assisted video monitoring, this study provides evidence on its potential to reduce inpatient falls and fall-related injuries, thereby addressing an important gap in the existing literature.

## 2. Materials and Methods

### 2.1. Study Design

This study employed a retrospective matched cohort design to evaluate the impact of AI-assisted video monitoring on inpatient falls by comparing the incidence rates of falls and fall-related injuries before and after its implementation.

### 2.2. Study Setting and Population

Data were collected from a tertiary hospital in C city, South Korea. The study included patients admitted to the hematology–oncology ward between 1 January 2020 and 31 December 2024. The observation periods were selected immediately before and after the system was implemented in 2022 to minimize potential changes over time in hospital policies and regulations, nursing practices, staffing, and infrastructure that could influence fall outcomes. Those admitted between 1 January 2020 and 31 December 2021 and 1 January 2023 and 31 December 2024 comprised the non-exposed and exposed groups, respectively. Although the AI-assisted video monitoring system was introduced in January 2022, the year 2022 was excluded from the analysis to allow for a transition period during which the staff became familiar with the system. Since January 2020, the ward has operated as a Comprehensive Nursing Care Unit (CNCU), where patient care is provided exclusively by registered nurses and nursing assistants, without the involvement of formal or informal caregivers.

The sample consisted of adult patients (≥18 years) admitted to multibed rooms. Since the video-monitoring system was implemented only in multibed rooms beginning in 2022, both the non-exposed and exposed groups were restricted to patients admitted to multibed rooms. Patients readmitted during the observation period were treated as separate independent cases. Patients admitted in late 2021 and discharged in 2022, or admitted in late 2024 and discharged in 2025, were also included in the non-exposed and exposed groups, respectively. As a result, 3393 admission cases were obtained from 1035 patients in the non-exposed group and 3315 admission cases were obtained from 1139 patients in the exposed group. To obtain a homogeneous distribution between the two groups, we used the nearest neighbor propensity score matching method for age, sex, the number of diagnoses and the number of ward days. This resulted in 1:1 matched data containing 3002 cases for each group.

### 2.3. Exposed Group and Technical Environment

The exposed group comprised patients admitted to the ward where the AI-assisted video monitoring was implemented. The system was Artificial Intelligence–Patient cAre Monitoring (AI-PAM; GeomexSoft, Chuncheon, Republic of Korea), which is an AI-assisted patient monitoring solution. AI-PAM consists of ceiling-mounted RGB cameras with fisheye lenses and an AI-driven program for action recognition and classification.

RGB cameras were installed in multibed rooms, specifically, two four-bed rooms and six five-bed rooms. Monitoring coverage was limited to these eight multibed rooms; single-occupancy rooms and areas outside patient rooms such as hallways were not covered by the AI-PAM system. The fisheye lenses provide a field of view greater than 180 degrees, thus minimizing blind spots. Video streams are continuously recorded and transmitted to a central server, which is connected to a separate network from external systems, and recorded videos are stored for only one day before being deleted for cybersecurity.

**Figure 1:**
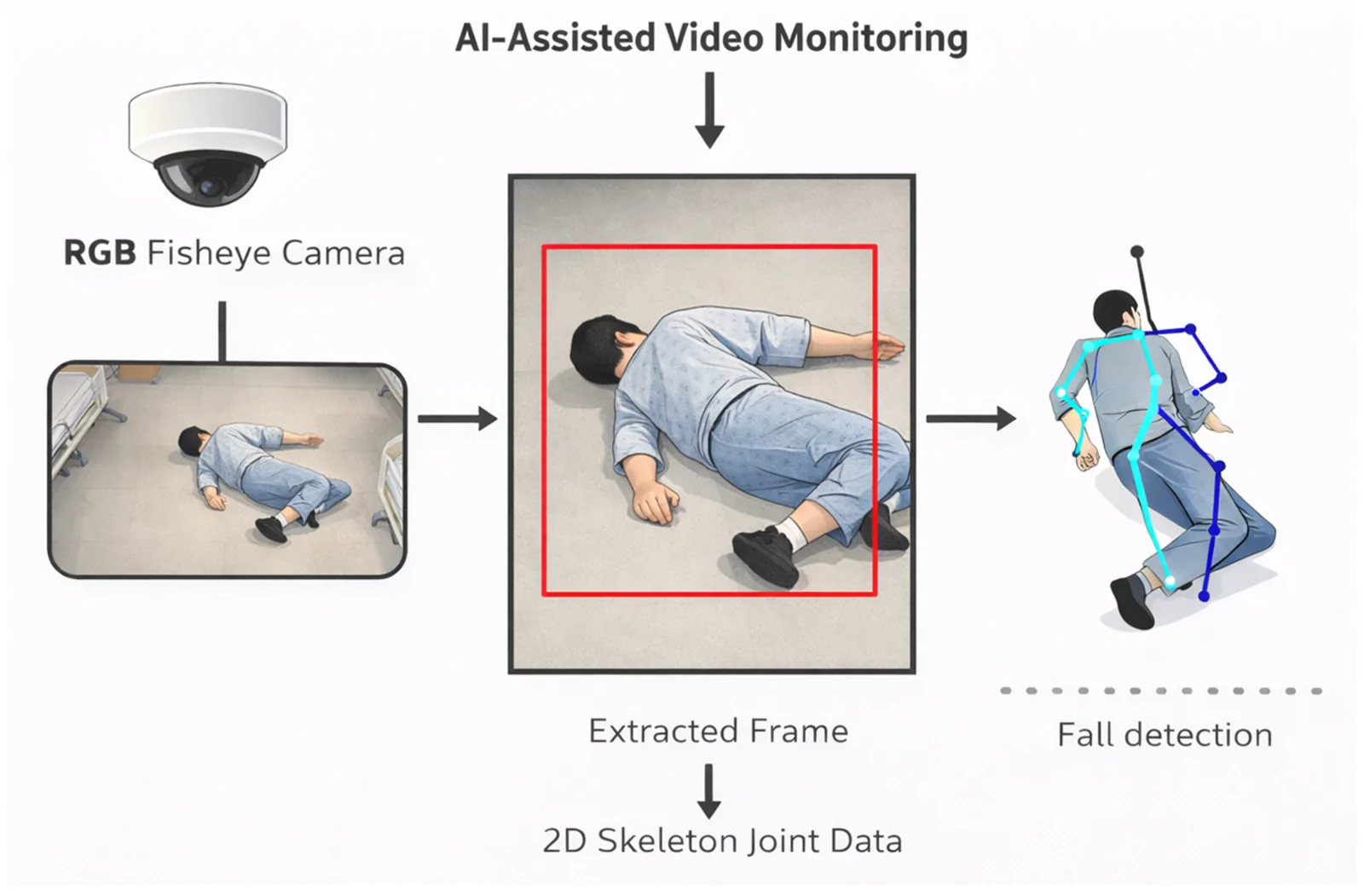
Overview of the AI-driven fall detection process, including frame extraction and two-dimensional skeleton-based movement analysis. This image was created with the assistance of ChatGPT (OpenAI), using GPT Image 1.5, for explanatory purposes and was reviewed and edited by the authors. It does not contain real patient data.

From these streams, frame sequences are extracted, and 2D skeleton joint data are computed from RGB images (Figure 1). Human pose and position are estimated using geometric and temporal features derived from skeleton sequences, and actions are classified as falls or non-falls in real time. This system utilizes the Panoptic DeepLab model, a convolutional neural network (CNN)-based deep learning architecture suitable for dense pose estimation and segmentation tasks [17]. Individual-level classification in shared rooms is achieved via object detection and tracking algorithms based on bed location. The system is programmed to send immediate alerts to the nurses’ station dashboard when it detects any of the following: (a) a patient lying on the floor, (b) a patient sitting on the floor, (c) squatting, or (d) bending at the waist. When an alert is triggered, the video window of the corresponding room automatically enlarges on the dashboard, thereby allowing nurses to verify the situation immediately. After reviewing the alert, nurses responded according to the patient’s condition and clinical circumstances, including visiting the patient’s bedside when immediate assessment or assistance was required.

In a performance test conducted by the Telecommunications Technology Association, the AI-PAM system demonstrated a fall detection rate of 99.7% for fall images and an alert latency of 0.0114 s from fall detection to alert generation [18]. However, sensitivity, specificity, and overall accuracy were not reported in the test results. The AI-PAM system continuously captured and analyzed video streams from the monitored rooms 24 h a day. When any of the aforementioned fall-related postures were detected, the system automatically generated an alert. Even in the absence of alerts, nurses can monitor patient conditions and room environments in real time through the dashboard, which supports simultaneous monitoring of all eight rooms. To protect patient privacy, video feeds are blurred to a level sufficient to recognize the situation. A “privacy mode” was activated when the curtains were closed to perform procedures, during which the video image was further blurred. An AI-based image generation tool was used to create a synthesized illustration for Figure 1 to aid in explaining the technical process; this image does not represent real patient data.

### 2.4. Non-Exposed Group

The non-exposed group included patients admitted to the same hematology–oncology CNCU prior to the implementation of the aforementioned system. 

### 2.5. Data Collection

Data on outcomes and covariates were collected via electronic medical records. Outcome variables included the occurrence of inpatient falls and fall-related injuries. An inpatient fall was defined as a fall occurring in the patient’s room during the ward stay. Falls that occurred after transfer to another ward or outside the patient’s room (e.g., in the hallway) were excluded because these areas were not covered by AI-PAM. Therefore, the observation area was restricted to monitored inpatient rooms where patients were directly exposed to AI-assisted video monitoring. The National Coordinating Council for Medication Error Reporting and Prevention’s 9-category index, which ranges from Category A (circumstances with the potential for error) to Category I (death), was used to evaluate a fall-related injury [19]. A fall-related injury was considered to have occurred when the severity level of the fall was reported as Grade E (temporary harm) or higher. To control for covariates, the following data were collected: age, sex, body mass index (BMI), the number of diagnosed diseases, the number of ward days, and Morse Fall Scale (MFS) score [20]. For propensity score matching and regression modeling, the number of diagnosed diseases and ward days were categorized based on their distributions and quartiles, and MFS scores were categorized according to established risk thresholds: low risk (<25), moderate risk (25–44), and high risk (≥45). All cases were removed if they were under 18 years of age, had no BMI, or had single- or triple-digit BMI values. All missing data for covariates were excluded from the analysis.

### 2.6. Statistical Analysis

The incidence rates of falls per 1000 patient days were calculated for each exposed and non-exposed group. In addition, the incidence rates of fall-related injuries with grade E or higher were computed. Chi-squared test, T-test and Fisher’s exact test were performed to compare the distribution of each covariate between the exposed and non-exposed groups. To verify the independence of each patient’s repeated hospitalization data, we performed a generalized estimating equation analysis by assuming a working correlation matrix as ‘exchangeable’ and ‘first-order autoregressive,’ respectively. As the estimated correlation coefficients were all very low (less than 0.001), we assumed that the data were independent. Conditional logistic regression and Cox proportional hazards regression were performed to assess the effectiveness of an AI-assisted video monitoring system. Conditional logistic regression was also performed to identify the association between each covariate and falls. Due to the small number of events in the 1:1 matched cohort, we applied Firth’s penalized likelihood logistic regression for fall and fall-related injury, incorporating a strata variable for the matched pairs to obtain robust odds ratios (ORs) and 95% confidence intervals (CIs) instead of standard conditional logistic regression. Firth’s penalized logistic regression is known to correct for statistical bias and prevent the error of overestimating risk due to an insufficient number of events [21,22]. For multiple regression modeling, Cox proportional hazards regression with robust standard errors was performed under the right-censoring scheme. The survival time was defined as the time from the day of admission to the ward to the day of fall. It was considered right-censored if no fall occurred by the day of discharge from the ward. The statistical software R 4.5.3 with the “logistf” package and “survival” package was used for the analysis [23].

### 2.7. Ethical Considerations

The study was conducted in accordance with the ethical principles of the Declaration of Helsinki. Approval for the study and use of data was obtained from the Institutional Review Board (approval no. KNUH-2025-05-002-001) and the Data Review Board (approval no. KNUH-2025-05-001) of a tertiary hospital in Chuncheon, Republic of Korea. Informed consent was waived because this was a secondary, retrospective study that did not involve the collection of new data. Patients admitted to camera-equipped rooms were informed about the purpose of video monitoring, use of the collected data, and data protection procedures through a privacy pledge provided prior to admission. They were informed that cameras would be installed in their rooms, except in bathrooms, and would remain visible at all times, and that audio recording was disabled. They were also informed that privacy protection was incorporated into the monitoring system, including image blurring and activation of a privacy mode during procedures. Written consent for video monitoring was obtained during the admission process.

## 3. Results

### 3.1. Comparison Between Exposed and Non-Exposed Groups

Among 3002 cases in each group, there were 17 fall events in the exposed group and 20 fall events in the non-exposed group. The fall incidence rate per 1000 patient-days in the group exposed to the AI-assisted video monitoring system was 1.017, which was lower than the rate of 1.286 in the non-exposed group. The exposed group had a significantly higher proportion of patients in the higher-risk MFS categories than the non-exposed group (χ^2^ = 80.113, *p* < 0.001) (Table 1).

### 3.2. Effects of AI-Assisted Video Monitoring on Inpatient Falls

The results of both univariate and multivariate penalized conditional logistic regression analyses showed no association between AI-assisted video monitoring and inpatient falls. Potential confounding variables that could not be included in the matching process were adjusted for in the multivariate penalized conditional logistic regression model. In the univariate analysis, the OR was 0.823 (95% CI, 0.445–1.617; *p* = 0.625), while in the multivariate analysis, after adjusting for other covariates such as BMI and MFS, the OR was 1.476 (95% CI, 0.649–3.596; *p* = 0.358) (Table 2). Although the adjusted OR was greater than 1 after adjustment for BMI and MFS, it was not statistically significant.

**Figure 2:**
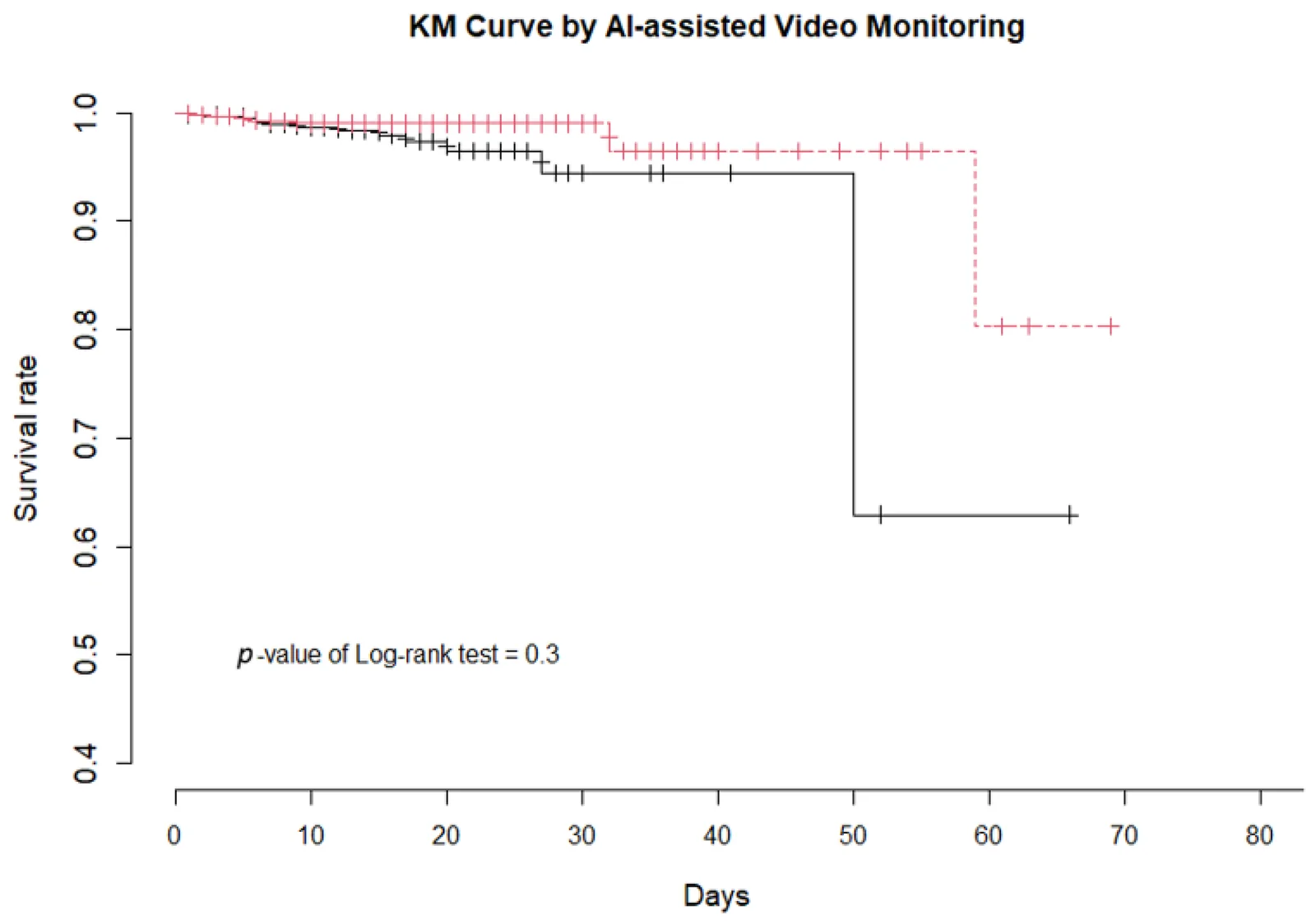
Kaplan–Meier curves of the exposed group (red) to the AI-assisted video monitoring system and the non-exposed group (black).

First, the Kaplan–Meier curve indicated that the fall-free survival rate in the exposed group was higher than that in the non-exposed group, but no significant difference was observed (log-rank test *p*-value = 0.3, Figure 2). The Cox proportional hazards regression model fit results revealed that no significant difference was established in the effect of AI-assisted video monitoring on falls (Crude HR, 0.717; 95% CI, 0.365–1.410), and the proportional hazards assumption was satisfied. The results of fitting a multiple Cox regression model, adjusted for other variables such as sex, age, BMI, the number of diagnoses, the number of ward days and MFS score, showed that AI-assisted video monitoring did not yield statistically significant results, consistent with the findings of the penalized conditional logistic regression analysis (Table 3).

### 3.3. Effects of AI-Assisted Video Monitoring on Inpatient Fall-Related Injuries

The incidence rates of fall-related injuries of grade E or higher per 1000 patient-days in the exposed and non-exposed groups were 0.359 and 0.257, respectively. In the exposed group, 17 falls were recorded, six of which resulted in grade E or higher injuries, while in the non-exposed group, four of 20 falls resulted in the same outcome. Fisher’s exact test based on data of fall patients showed that there was no significant association between fall-related injury and AI-assisted video monitoring (OR, 2.135; 95% CI, 0.396–12.925; *p* = 0.459) (Table 4). These results were also confirmed in a penalized conditional logistic regression analysis performed on all patients after adjusting for BMI and MFS (OR, 3.207; 95% CI, 0.634–31.431; *p* = 0.168) (Table 5).

## 4. Discussion

This study sought to examine the effect of AI-assisted video monitoring on inpatient falls and fall-related injuries. Given the limited literature on AI-assisted video monitoring systems, our findings provide valuable evidence to support the development and broader adoption of AI-assisted fall prevention systems in clinical practice.

The evaluated system enabled real-time monitoring, visual verification, and prompt response to alarms and was therefore expected to reduce fall events and fall-related injuries. Although fall incidence decreased from 1.286 to 1.017 per 1000 patient-days following the implementation of AI-assisted video monitoring, logistic and survival regression analyses indicated this reduction was not statistically significant. Previous studies have reported mixed findings. Hogan Quigley et al. (2022) reported a reduction in fall rates (3.93–3.37 per 1000 patient-days) and fall-related injuries (0.95–0.89 per 1000 patient-days) after video monitoring, although statistical significance was not reported [10]. Similarly, Ergai et al. (2024) found a significant 39% decrease in falls with injuries (0.648–0.394 per 1000 patient-days) in the six months post-implementation, while no change in overall fall incidence [11]. While our study used an AI-driven alarm system, these studies adopted video monitoring by a human virtual sitter, which exhibited some preventive elements in reducing falls. Despite the challenges of communication between virtual sitters and nurses, virtual sitters may help prevent falls by detecting early signs of potentially hazardous behaviors, so-called “nuisance” behaviors, before an actual fall occurs, thus functioning as an effective preventive strategy [10,11]. Although our system was capable of alerting nurses to actual or potential falls by detecting specific postures and positions, such as lying on the floor, it may be limited as a proactive preventive strategy. More recently, Tyransky et al. (2025) reported a 59% reduction in falls using a virtual sitter model supported by AI alerts, which also reduced sitter hours and bedside nurse workload [24]. In that system, AI support provided visual cues regarding changes in patients’ center of gravity to the virtual sitter, which likely reduced sitter cognitive load and minimized the risk of human error. These findings suggest that future research should identify and validate optimal sitter models that incorporate AI to detect fall risks in advance and better support clinical nursing workflow.

To enable AI-assisted video monitoring to function as a truly preventive intervention, systems must detect risky behaviors and environmental cues that signal increased fall risk and generate timely alerts—similar to the role virtual sitters play in detecting hazardous behaviors and notifying nurses. For example, recognizing attempts to climb over bed rails could facilitate rapid nursing intervention before an actual fall occurs. A recent study by Gervasi et al. (2025), which evaluated an AI-assisted system similar to ours, demonstrated that detecting risky situations such as attempts to leave the bed, bed exits, prolonged toilet stay, and room exits, in addition to actual falls and delivering alerts to nurses via tablet devices led to a reduction in inpatient fall rates [25].

Achieving this preventive capability will require further technical refinement to improve the accuracy of detecting patient behaviors and environmental conditions. Although deep learning-based fall detection systems have achieved accuracies exceeding 90%, and in some cases approaching 100%, these results have largely been obtained using datasets collected in controlled laboratory environments [26]. Their performance may not generalize to real-world clinical settings, where occlusion, scale variation, and the diversity of patient behaviors present substantial challenges. In the present study, the AI-PAM system demonstrated a fall detection rate of 99.7% in a performance test. However, sensitivity, specificity, and overall accuracy were not reported, and the reported detection rate was therefore not directly comparable with the performance measures used in previous studies. This limitation should be considered when interpreting the system’s performance and the study findings. Further validation using standardized performance measures in clinical settings is warranted. In addition, fall-related movements are infrequent and often overlap with normal daily activities, leading to class imbalance that limits the performance and generalizability of deep learning models [27]. Frequent false alarms may also increase nurses’ workload and contribute to alarm fatigue, reducing the practical usefulness of the system. Accuracy is expected to improve through the integration of multiple sensors, the accumulation of larger and more diverse datasets, and continued advances in deep learning-based human activity recognition (HAR) [28]. Such technological refinement may enhance the clinical utility of AI-assisted video monitoring as a proactive fall prevention strategy.

AI-assisted video monitoring may reduce fall-related injuries through several mechanisms, including prevention of falls by real-time monitoring of risky behaviors or environment, earlier detection of falls, and facilitation of timely nursing responses. In the present study, however, the incidence rate of fall-related injuries was numerically higher in the exposed group than in the non-exposed group (0.359 vs. 0.257 per 1000 patient-days), although the difference was not statistically significant. Similarly, the proportion of falls resulting in injury was higher in the exposed group (0.353 vs. 0.2). The reasons for these differences could not be determined, particularly given the small number of fall events and fall-related injuries. In addition, clinical indicators such as nursing response time following fall alerts were not available in the secondary data. Previous research has shown that AI-assisted video monitoring can shorten staff response times after fall events [12]. Early detection and timely intervention may contribute to mitigating the severity of fall outcomes. Therefore, future prospective studies should incorporate measures of early detection and nursing response time to more comprehensively evaluate the clinical value of AI-assisted video monitoring.

This study has several limitations. First, the exposed and non-exposed groups were not fully comparable in MFS score. This imbalance may have influenced the outcomes and thus represents a limitation of this study. This is because the high rate of missing values in the MFS variable prior to the matching stage limited its utility as a matching variable. Instead, we adjusted for this within the multivariate regression models. However, the high proportion of missing MFS data may have influenced the multiple regression analysis results.

Second, as with any before-and-after study design, the possibility of temporal confounding cannot be excluded. To minimize potential influence of changes in hospital policies and regulations, nursing practices, staffing, and hospital infrastructure, we selected observation periods immediately before and after the implementation of AI-assisted video monitoring, excluding 2022 as a transition period. The study ward remained under the same clinical department throughout the observation periods, with no notable changes in hospital policies or regulations, nursing practice, staffing structure, or infrastructure. However, nursing practices in the unexposed period may have been affected by the COVID-19 pandemic, including restrictions on visits by family caregivers and transfers to isolation wards for patients with COVID-19. These changes may have influenced patient monitoring and fall-prevention practices and, consequently, the study outcomes. In addition, the Hawthorne effect cannot be excluded, as staff awareness of the implementation of the AI-assisted video monitoring may have influenced their fall-prevention practices. However, the 1-year transition period was excluded from the observation periods to allow staff to become familiar with the system.

Third, the retrospective design limited our analysis to existing data, restricting a comprehensive evaluation of the AI-assisted video monitoring system. Fall events were identified solely through institutional incident reports, raising the possibility that some unwitnessed or unreported falls were not captured. Previous research has suggested that video monitoring may increase the detection of previously unrecognized falls [9]. Another limitation is that some potentially relevant fall-risk factors were not routinely recorded and therefore could not be assessed or adjusted for. For example, footwear type, which may influence gait stability and fall risk [29,30], was not available in the study data. This may have resulted in residual confounding.

Fourth, this study was conducted in a single hospital ward, specifically, a CNCU where patients with substantial cognitive impairment, mobility limitations, or those requiring sedative or psychoactive medications were generally not admitted. Thus, the study population generally comprised patients with relatively lower fall risk. Consequently, the number of fall events was limited, which potentially reduced the statistical power of our analyses. To mitigate potential bias and improve the statistical performance associated with the small number of events, Firth’s penalized logistic regression and Cox proportional hazards regression with robust standard errors were used. Nevertheless, the limited number of fall events remains a limitation of the study. In addition, the findings may not be generalizable to higher-risk patient populations. To enhance the robustness and generalizability of the findings, future research should include larger and more diverse patient populations.

Finally, this study did not evaluate the system’s usability or impact on nursing workflow in real clinical practice. Therefore, future research should investigate nurses’ utilization and perceptions of the system, develop fall prevention management algorithms that optimize system use, and ultimately conduct rigorous evaluations of the effectiveness of AI-assisted video monitoring in preventing falls.

## 5. Conclusions

This study evaluated the association between AI-assisted video monitoring and inpatient falls. Although a lower fall incidence was observed during the exposed period than during the non-exposed period, this difference was not statistically significant. Given the retrospective before-and-after study design and the use of secondary data, it was difficult to adequately control for potential confounding factors. In addition, the small number of fall events may have limited the statistical power of the analyses. Therefore, prospective studies with adequate sample sizes and appropriate control of potential confounders are needed to further evaluate the effects of AI-assisted video monitoring on inpatient falls. Further development of optimal sitter models that incorporate AI for detecting fall risks, along with continued technical refinements to identify risky behaviors and environmental cues, will be important for realizing the potential of AI-assisted systems to support proactive fall-prevention interventions. Future research should comprehensively assess the value of AI-assisted video monitoring, including usability, impacts on nursing workflow, and clinical effects across diverse patient populations.

## Figures and Tables

**Table 1:** Characteristics of matched data with respect to sex and age, the number of diagnoses, and the number of ward days.

Covariate	*n*	Frequency (Column Proportion) or Mean (Standard Deviation)		Test Statistics and *p*-Value from Chi-Squares or *t*-Test
		Non-Exposed Group	Exposed Group	
Incidence rate of falls per 1000 patient-days		1.286	1.017	
Sex				$\chi^{2}$ = 0, *p* = 1
male	3331	1666 (0.55)	1665 (0.55)	
female	2673	1336 (0.45)	1337 (0.45)	
Age				$\chi^{2}$ = 6.686, *p* = 0.153
18–49	351	185 (0.06)	166 (0.05)	
50–59	886	411 (0.14)	475 (0.16)	
60–69	1911	965 (0.32)	946 (0.32)	
70–79	1612	803 (0.27)	809 (0.27)	
>=80	1244	638 (0.21)	606 (0.20)	
BMI				$\chi^{2}$ = 5.089, *p* = 0.165
<18.5	825	385 (0.13)	440 (0.15)	
18.5–22.9	2576	1316 (0.44)	1260 (0.42)	
22.9–24.9	1143	577 (0.19)	566 (0.19)	
>=24.9	1460	724 (0.24)	736 (0.24)	
No. of diagnoses				$\chi^{2}$ = 1.119, *p* = 0.572
1	5044	2507 (0.84)	2537 (0.85)	
2	882	455 (0.15)	427 (0.14)	
>=3	78	40 (0.01)	38 (0.01)	
NA	20			
No. of ward days				
1–3	3509	1733 (0.58)	1776 (0.59)	$\chi^{2}$ = 3.998, *p* = 0.136
4–14	2122	1094 (0.36)	1028 (0.34)	
>=15	373	175 (0.06)	198 (0.07)	
MFS				
0–24	3761	1857 (0.62)	1904 (0.63)	$\chi^{2}$ = 80.113, *p* < 0.001
25–44	733	281 (0.09)	452 (0.15)	
45–125	176	36 (0.01)	140 (0.05)	
NA	1334	828 (0.28)	506 (0.17)	

**Table 2:** Results of univariate and multivariate penalized conditional logistic regression analyses regarding fall in the matched data.

Covariate	*n*	Frequency (Proportion)		Univariate		Multivariate ^‡^	
		Fall	No Fall	OR (95% CI ^†^)	*p*-Value	OR (95% CI)	*p*-Value
Video Monitoring					0.625		0.358
Exposed	3002	17 (0.006)	2985	0.823 (0.445, 1.617)		1.476 (0.649, 3.596)	
Non-Exposed	3002	20 (0.007)	2982	Reference		Reference	
BMI							
<18.5	825	6 (0.007)	819	Reference		Reference	
18.5–22.9	2576	15 (0.006)	2561	0.763 (0.317, 2.062)	0.571	0.865 (0.306, 2.913)	0.798
22.9–24.9	1143	7 (0.007)	1136	0.832 (0.287, 2.479)	0.733	0.616 (0.136, 2.562)	0.498
>=24.9	1460	9 (0.006)	1451	0.825 (0.306, 2.367)	0.709	0.944 (0.284, 3.440)	0.927
MFS							
0–24	3761	15 (0.004)	3746	Reference		Reference	
25–44	733	7 (0.009)	726	2.495 (0.980, 5.810)	0.055	2.021 (0.767, 4.891)	0.147
45–125	176	2 (0.011)	174	3.463 (0.682, 11.294)	0.118	2.480 (0.465, 8.746)	0.250
NA	1334						

^†^ CI = confidence interval ^‡^ Age was included as a control factor in the multivariate analysis model to adjust for the imbalance caused by missing values in MFS.

**Table 3:** Crude and adjusted hazard ratios from Cox proportional hazards regression models with matched data.

Covariate	*n*	Frequency (Proportion)		Crude HR ^†^ (95% CI ^‡^)	Adjusted HR (95% CI)
		Fall	No Fall		
Video Monitoring					
Exposed	3002	17 (0.006)	2985	0.717 (0.365, 1.410)	1.334 (0.537, 3.314)
Non-Exposed	3002	20 (0.007)	2982	Reference	Reference
Sex					
male	3331	20 (0.006)	3311	Reference	Reference
female	2673	17 (0.006)	2656	1.011 (0.532, 1.992)	1.591 (0.685, 3.696)
Age					
18–59	1237	4 (0.003)	1233	Reference	Reference
60–69	1911	11 (0.006)	1900	1.916 (0.625, 5.879)	1.574 (0.438, 5.660)
70–79	1612	10 (0.006)	1602	1.982 (0.634, 6.197)	1.907 (0.553, 6.583)
>=80	1244	12 (0.010)	1232	2.442 (0.789, 7.554)	1.910 (0.299, 12.212)
BMI					
<18.5	825	6 (0.007)	819	Reference	Reference
18.5–22.9	2576	15 (0.006)	2561	0.993 (0.387, 2.551)	1.119 (0.369, 3.399)
22.9–24.9	1143	7 (0.007)	1136	1.089 (0.364, 3.258)	0.802 (0.185, 3.466)
>=24.9	1460	9 (0.006)	1451	0.934 (0.334, 2.611)	0.965 (0.284, 3.275)
No. of diagnoses					
1	5044	25 (0.005)	5019	Reference	Reference
2	882	10 (0.011)	872	1.377 (0.649, 2.921)	1.064 (0.379, 2.986)
>=3	78	2 (0.026)	76	3.50 (0.816, 15.016)	2.507 (0.336, 18.723)
NA	20				
No. of ward days					
1–3	3509	7 (0.002)	3502	Reference	Reference
4–14	2122	9 (0.004)	2103	2.009 (0.766, 5.273)	3.099 (0.931, 10.316)
>=15	373	11 (0.030)	362	1.476 (0.358, 6.092)	1.508 (0.186, 12.242)
MFS					
0–24	3761	15 (0.004)	3746	Reference	Reference
25–44	733	7 (0.009)	726	1.846 (0.725, 4.702)	1.412 (0.547, 3.645)
45–125	176	2 (0.011)	174	1.355 (0.254, 7.230)	0.888 (0.121, 6.534)
NA	1334				
Likelihood ratio test					$\chi^{2}$ = 9.89, *p* = 0.7701

^†^ HR, hazard ratio; ^‡^ CI, confidence interval.

**Table 4:** Results for fall-related injury in fall patients.

Covariate	*n*	Frequency (Proportion)		Fisher’s Exact Test	
		Injury	No Injury	OR ^†^ (95% CI ^‡^)	*p*-Value
Video Monitoring				2.135 (0.396, 12.925)	0.459
Exposed	17	6 (0.353)	11		
Non-Exposed	20	4 (0.2)	16		

^†^ OR, odds ratio; ^‡^ CI, confidence interval.

**Table 5:** Results of univariate and multivariate penalized conditional logistic regression analyses regarding fall-related injury in the matched data.

Covariate	*n*	IR ^†^	Frequency (Proportion)		Univariate		Multivariate ^‖^	
			Injury	No Injury	OR ^‡^ (95% CI ^§^)	*p*-Value	OR(95% CI)	*p*-Value
Video Monitoring						0.545		0.168
Exposed	3002	0.359	6 (0.002)	2996	1.445 (0.438,5.225)		3.207 (0.634,31.431)	
Non-Exposed	3002	0.257	4 (0.001)	2998	Reference		Reference	

^†^ IR, Incidence rate of fall-related injury per 1000 patient-days; ^‡^ OR, odds ratio; ^§^ CI, confidence interval. **^‖^** Multivariate penalized regression was fitted, adjusted for age, BMI, and MFS.

## Data Availability

The datasets analyzed during the current study are not publicly available due to privacy and ethical restrictions. Summary data supporting the findings of this study are provided within the manuscript.

## Abbreviations

The following abbreviations are used in this manuscript:

AI	Artificial intelligence
CNCU	Comprehensive Nursing Care Unit
AI-PAM	Artificial Intelligence–Patient cAre Monitoring
RGB	Red-green-blue
CNN	Convolutional neural network
BMI	Body mass index
MFS	Morse Fall Scale
OR	Odds ratio
CI	Confidence interval
HR	Hazard ratio
IR	Incidence rate
HAR	Human activity recognition
